# Dental Fiber-Post Systems: An In-Depth Review of Their Evolution, Current Practice and Future Directions

**DOI:** 10.3390/bioengineering10050551

**Published:** 2023-05-04

**Authors:** Abdulrahman Alshabib, Khaled Abid Althaqafi, Hani S. AlMoharib, Mahir Mirah, Yasser F. AlFawaz, Hamad Algamaiah

**Affiliations:** 1 Department of Restorative Dental Science, College of Dentistry, King Saud University, Riyadh 11545, Saudi Arabia; 2 Department of Restorative Dental Department, College of Dentistry, University of Umm Al Qura, Makkah 24211, Saudi Arabia; 3 Department of Periodontics and Community Dentistry, College of Dentistry, King Saud University, Riyadh 11545, Saudi Arabia; 4 Department of Restorative Dental Science, Dental College, and Hospital, Taibah University, Madinah 42353, Saudi Arabia

**Keywords:** restorative dentistry, dental fiber-post, dental post, dental materials, dental restoration, endodontically treated teeth, fiber-reinforced composite, prosthodontics, EndoCrown, indirect restoration, single crowns, Resin cement, adhesive dentistry

## Abstract

The field of dental medicine is constantly evolving and advancing toward minimally invasive techniques. Several studies have demonstrated that bonding to the tooth structure, particularly enamel, yields the most predictable results. In some instances, however, significant tooth loss, pulpal necrosis, or irreversible pulpitis may limit the options available to the restorative dentist. In these cases, placement of a post and core followed by a crown is the preferred treatment option, provided all requirements are met. This literature review provides an overview of the historical development of dental FRC post systems as well as a comprehensive examination of the currently available posts and their bonding requirements. In addition, it offers valuable insights for dental professionals seeking to understand the current state of the field and the prospects of dental FRC post systems.

## 1. Introduction

The susceptibility of endodontically treated teeth (ETT) to fracture has long been a concern in dentistry. Initially, it was believed that changes in the tooth dentine, such as reduced moisture content and decreased collagen cross-linking, were the leading causes of ETT fractures [1,2]. However, ETT fractures are primarily caused by changes in tooth structural integrity [3,4]. Access preparation can result in structural integrity loss and increased cuspal deflection, which increases the risk of microleakage at restoration margins and tooth fractures [5]. Additionally, ETTs are more vulnerable to fractures due to the lack of proprioception. Nevertheless, Schneider et al. [6]. reported that vital and non-vital teeth have comparable tactile sensitivity. These factors contribute to making ETT final restorations a crucial consideration.

The demand for tooth-colored posts has increased the use of non-metallic posts. The development of dental materials has fundamentally altered the concept of post-and-core systems, allowing for a shift from mechanically retained restorations to adhesively retained ones. As non-metallic materials become more prevalent in indirect restorations, the role of the ferrule becomes less significant, and the properties and performance of the adhesive systems and root canal posts are now of paramount importance. The adhesive interface between the post and dentin must be sufficiently strong to transmit the load from the occlusal surface to the periodontal ligaments and root. Zirconia posts, epoxy or methacrylate resin posts reinforced with quartz or glass fibers, polyethylene fiber-reinforced epoxy resin posts, and epoxy resin posts reinforced with carbon fibers are the most commonly used posts [1].

All of these advancements and material options can complicate clinical decision-making regarding ETT replacement, including whether or not to post, as well as what materials to use. Furthermore, a few published reviews of fiber posts discussed the physio-mechanical properties and associated techniques of fiber posts. Therefore, this literature review aims to provide a comprehensive summary of the factors that influence the use and selection of FRC to restore ETT. In addition, this study intends to assess the current alternatives and future prospects for ETT restorations.

## 2. History of Dental Fiber Posts

According to their manufacturers, FRC posts were introduced to clinical practice for the first time in France in 1989. Duret published the first research article on dental fiber posts in 1990 [2]. Due to their suitable mechanical properties, such as tensile strength and stiffness, electrical conductivity, and low toxicity, carbon/graphite fibers were chosen for this application [3]. This material revolutionized dentistry since it was an efficient alternative to metal posts. The modulus of elasticity of these fiber-reinforced materials was closer to that of dentin than the previously used metal posts, and the results of clinical studies were promising [4]. Since their introduction, they have gained popularity among dental clinicians due to their reliable clinical performance and variety of advantages, such as their mechanical, esthetic, and elastic properties. However, the initial posts did have a disadvantage in terms of esthetics, as they were still visible beneath restorations made entirely of ceramic or composite. With the incorporation of quartz and glass fibers into the resin, more radiopaque fiber posts have been developed over time. This rendered them translucent or white and therefore more aesthetically pleasing. In addition, they have been widely adopted due to their additional benefits, which include high tensile strength, resistance to biochemical degradation and solubility, low electrical conductivity, and low electrical conductivity.

## 3. Longevity and Failure of Dental Fiber Posts 

Although fiber posts have demonstrated good physical and mechanical properties in vitro, numerous failures have been reported in clinical practice [5,6,7,8,9,10,11,12,13,14,15,16,17,18,19,20,21,22]. This demonstrates the limitations of the current fiber post materials. Table 1 summarizes clinical studies demonstrating the failure rate associated with fiber posts in clinical practice.

The reported failure rate of ETTs with fiber-reinforced posts ranged from 2% to 40%. This wide range can be attributed to a number of variables, including (i) variability of teeth restored (anteriors vs. premolars vs. molars), (ii) anatomical variability for root canals, (iii) variability of statistical significance (study power analysis, sample size), (iv) variability of luting cement, and finally (v) variables in final restorations (direct vs. indirect). 

Post-debonding failures were the most prevalent, followed by endodontic failures [8,17,18]. In general, post-related failure rates were lower than the actual reported failure rates. For instance, Ferrari et al. reported a failure rate of 7–11%, of which only 2.3% were attributed to post-failure [14]. Similarly, Mannucci et al. [9] study had a 2.5% post-related failure compared to the reported 3.8%.

Tooth type and remaining tooth structure (remaining walls) are the primary determinants of tooth longevity [15,16,17]. Cox regression analysis regarding tooth type revealed a significantly higher hazard ratio of 2–2.5 for posterior teeth [12,16,17]. Therefore, longevity of ETT in molars is higher than that in premolars and anterior teeth [16,17,21]. Moreover, the presence of one or fewer remaining walls was significantly associated with higher failure rates [15,17].

There were no significant differences in longevity between cast and fiber-reinforced posts; however, more unrepairable failures necessitated extractions [13,16]. In contrast, failed ETT with fiber-reinforced posts showed more repairable failures (up to 85%) [20].

The type of final restoration had an effect on the long-term success of ETT. Direct restoration, such as extensive amalgams and crowns, is similar if amalgam restoration was designed to protect undermined cusps with good resistance and retention forms. Full coverage restoration with enough remaining tooth structure and Furrel influenced the longevity more than the type of post used [21]. Self-adhesive resin cement for post-cementation demonstrated a statistically insignificant advantage over conventional cement [18,21] in terms of bond strength [18,21].

## 4. Composition of a Fiber Post 

Several crucial properties of fiber composites, such as their optical, mechanical, and bonding properties, are determined by the type of fiber reinforcement and matrix used, in addition to the adhesion between their interfaces. Both the fibers and the matrix contribute in different ways to the stability of the material. The fibers provide stiffness and strength, while the polymeric matrix ensures the integrity of the composite structure and protects the fiber from damage due to high humidity and temperature. The matrix also distributes and transfers the load between the fibers. FRCs have been successfully employed in dentistry for many applications, such as orthodontic and periodontal splints, fixed partial dentures (FPDs), and removable prosthodontics [23].

FRCs can have either a thermoplastic (linear) or thermoset (cross-linked) matrix. The latter is most utilized in FRC canal posts and contains dimethacrylate or multifunctional resins, such as epoxy resin and bisphenol A-glycidyl methacrylate (Bis-GMA) and urethane di-methacrylate (UDMA). With their highly cross-linked networks, thermosetting polymers have superior mechanical properties, durability, thermal stability, and chemical resistance to thermoplastics; however, their surface adhesive properties are inadequate [24]. This is due to the resin in the luting cement or the core materials, which tend to swell or dissolve the polymers, negatively impacting the bond with the thermosetting matrix of FRC post [25]. This highly cross-linked structure also makes it difficult to remove the post if nonsurgical endodontic retreatment is required [26].

The fiber accounts for the largest proportion of FRCs (40–65 vol.%); therefore, the fiber determines the load-bearing capacity of the structure and significantly contributes to the matrix strength and stiffness [27]. Dental FRCs are reinforced with a variety of fibers, such as polyethylene, carbon, glass, and Kevlar (p-phenylene diamine). They are utilized with either uni-directional or woven orientation.

The effectiveness of the reinforcing role of fibers is dependent on a variety of factors, including their orientation, diameter, compatibility, quantity, and impregnation with the matrix resin [28]. For fibers to be effective in stress transmission and reinforcement, it is essential that the matrix saturate them with water; this also determines their mechanical properties and water absorption capacity. The adhesion between the fiber and matrix depends on the interaction between the two components, which may be chemical or mechanical. The degree of mechanical bonding depends on the surface texture and morphology of the fiber, whereas a coupling agent can be used to create a chemical covalent bond. Silanization of the fiber can improve its wettability, and the formation of hydrogen bonds and siloxane bridges on the fiber’s surface can improve its adhesion [28].

## 5. Biomechanical Considerations for FRC Posts

### 5.1. Stress Distribution

Researchers and clinicians have been interested in the biomechanical changes that occur in ETTs restored with posts and cores for several years. For the past decades, cast posts were utilized, but this began to change with the introduction of FRC posts and increased demand [29]. Despite their popularity, the biomechanical properties of FRC posts and their associated tooth behavior remain controversial [30].

The main purpose of the post is to preserve the integrity of a coronal restoration on a tooth with extensive loss of coronal structure [31,32]. In order for the post to effectively serve its intended purpose, it is crucial to understand the biomechanical factors that affect its longevity [32]. Therefore, research has been carried out using finite element analysis (FEA) and photoelasticity [33] to examine various post systems and their associated stress levels in endodontically treated teeth. Once bonding has occurred between the post and the tooth’s root canal, the biomechanical behavior of the former changes significantly [34]. Therefore, the material composition (zirconia, fiber, gold, quartz, titanium, or stainless steel) determines the concentration and distribution of stresses. The significance of analyzing stress lies in the fact that if stress is highly concentrated in a particular area, the risks of the tooth fracturing and the bonds between interfaces being broken increase.

In a maxillary central incisor during occlusion, the most damaging areas of stress concentration are the middle third of the root canal and the external cervical area of the tooth [35]. During occlusion, stress occurs in the root’s external coronal section below the clinical crown. Conversely, after inserting a post, greater stress levels were observed at the point where the tooth and post came into contact, which was at the internal buccal plate of the root. The materials that resulted in the lowest stress levels at this point were glass fiber and carbon fiber [35]. When stress is concentrated around the post, there is also increased stress at the adhesive interface. This may threaten the bond affecting the longevity of the restoration. Therefore, the post-dentin-bonded interface is critical concerning the concentration of stress. The risk of root fracture is higher in cases of weakened tooth structure, especially if dental work involves unnecessary removal of sound tooth structure [36].

The survival of FRC posts that have been adhesively luted is good, attributed to the similarity in the mechanical behavior of FRC posts and natural tooth structure [37]. If the post and core are too stiff (e.g., stainless steel), loading stresses will increase, increasing the likelihood of catastrophic tooth or restoration fracture [38]. The likelihood of root fracturing is reduced when using FRC posts, and in case of failure, they are often repairable [39]. 

### 5.2. Influence of Ferrule on Fracture Resistance 

The term ‘ferrule’ is believed to be a combination of ‘viriola’, which is Latin for a small bracelet, and ‘fer-rum’ (iron). It tends to be made from metal and it is an encircling band or clamp that is used to reinforce, join or fasten posts, wires or fibers. It is defined in dentistry as a “360-degree metal collar of the crown surrounding the parallel walls of the dentin extending coronally to the shoulder of the preparation. The result is an increase in resistance form of the crown from the extension of dentinal tooth structure”. Its main purposes are to prevent fracture and increase resistance to dislodgment [40]. It is often misused to mean the quantity of sound dentin that persists above the finish line, but in reality, the ferrule effect is the bracing of the whole crown over the tooth structure above the preparation margin.

The ferrule is essential for the stability of restored teeth that have been treated endodontically and, therefore, for their prognosis [41]. Nonetheless, it should be remembered that the restoration of an endodontically treated tooth involves a complex process, of which the ferrule effect is just one element. Several other factors affect the clinical performance of the restorative complex, such as the material used for the post and core, the overlying crown, the luting agent, and the functional occlusal load [38].

One of the fundamental requirements for a stable restoration is ensuring that there is sufficient dentin height. Fracture resistance and the number of cycles required before the restoration fail to increase with ferrule height [42,43]. Some studies have reported that a minimum height of 1 mm remaining tooth structure is sufficient [40,43]. However, others have found that a better performance is achieved in the long term with 1.5–2 mm or more [44,45]. Some researchers have stated that a ferrule appears to offer no advantage [46,47], but it does seem to result in more favorable fracture patterns. Moreover, if a fracture occurs in a tooth that does not contain a ferrule, it is most likely non-restorable.

Another significant factor in fracture resistance is the ferrule width. This is the thickness of the coronal extension above the crown margin [48]. If preparation for the restoration is extensive due to esthetic requirements or large caries lesions, this can drastically affect the buccal wall’s thickness. However, clinical practice does not recommend less than 1 mm dentin wall thickness [48,49]. However, walls of this thickness are more prone to fracturing than those with a thickness of 2 or 3 mm [50]. With this in mind, it is essential that dentin walls be preserved as much as possible and that the preparation for post and core buildup be executed with minimum invasiveness. This is essential to achieve an adequate ferrule. 

Research has shown that the performance of a homogeneous ferrule with an even circumference is higher than that of a heterogeneous one that is not equal in circumference over all parts of the tooth [51,52,53,54]. However, achieving a circumferential ferrule that is the same height throughout can be challenging and sometimes impossible. A uniform circumferential 2 mm ferrule will prevent failure better than one that is not uniform, for example ranging between 0.5 mm proximal and 2 mm buccal and lingual, or a 2 mm ferrule that covers only the buccal or palatal buccal part of the tooth, or even a discontinuous ferrule caused by bi-proximal cavitations [52,54]. However, an uneven ferrule is better than no ferrule at all [53,54]. On the other hand, in a restored tooth with no ferrule, the survival rate is better if there is only a buccal or palatal wall [55,56]. In order to deliver an adequate ferrule, it may be necessary to employ techniques such as orthodontic forced tooth eruption or crown lengthening [48]. Therefore, a minimum of 2 mm ferrule should be present on the lingual and buccal walls. 

### 5.3. Long or Short Post

Regardless of the material used to fabricate the post, the lengthier they are, the longer they will survive [57]. There is a proportional relationship between frictional retention and the contact area; the greater the contact area, the higher the retention level. This finding explains the results of the macro push-out and pull-out tests in which the entire post became detached.

Fracture resistance is also affected by the post length; however, there is no conclusive evidence on this issue. The biomechanical performance of cast posts and cores versus stainless steel and fiber posts was not affected by post length, according to a number of studies [39]. Zicari et al. studied short fiber posts used in ETT and reported that they could withstand fatigue similarly to long fiber posts [58]. According to the same study, failures in teeth with short posts may be more amenable to repair, allowing for reintervention and tooth preservation [58]. Short posts can also be more resistant to fractures due to their less invasive buildup approach.

In contrast to the previous findings, Giovani et al. [39] discovered that the fracture resistance of 10-mm-long posts was greater than that of 6-mm-long posts. Buttel et al. study assessed posts that were just 3 mm long against those of 6 mm; the posts underwent cyclic fatiguing in a chewing simulator, and the longer posts outperformed the shorter ones [59]. Therefore, they concluded that a minimum of 6 mm post length was to be used. This indicates that clinicians must carefully evaluate the length of posts for each case they deal with, considering the thickness of the remaining dentin, the concentration of stress, the bone support surrounding the root and the suggested type of restorative treatment. 

Since the anatomic complexity is greater in the apex and there are numerous lateral and access canals, how much of the remaining root canal filling material is a crucial factor [60]. Apical periodontitis cases are low in teeth that have been endodontically restored and which have at least 5 mm gutta-percha remaining in the apex [61]. It is important to avoid gaps between the root canal filling and the apical tip of the post because this can lead to periapical pathosis. These gaps can have a substantial impact on the success of endodontic treatment [61].

### 5.4. Post Space and Cement Thickness 

The fit and retention of the primary post are evaluated using pilot drills, through which a form-congruent root canal is created up to the apical third of the root, per standard clinical protocols for post-placement. This ‘form-congruence’ is intended to adapt the post to the surrounding root canal walls utilizing a thin and even layer of post-root cement [62]. If the fit between the post and the root canal is good, stress will be more evenly distributed along the canal wall during clinical function [63]. The retention of prefabricated, non-adhesive-cemented posts is diminished proportionally to the fit between the post and canal.

If a tooth’s canals are oval or irregular in shape, the post space must be meticulously reshaped to produce a round and form-congruent shape. This necessitates the removal of a substantial amount of inner dentin, which weakens the tooth and reduces its resistance to fracture. If there is no form congruence in the canals, it is necessary to use oval posts and preparation tips to avoid excessive tooth reduction [64]. Posts are selected to preserve the inner dentin structure, which necessitates minimal preparation and a high degree of correspondence with the actual root canal diameter. 

### 5.5. Thick or Thin Post

In regard to metal posts, the factor that appears to have the most significant effect on fracture resistance is the post diameter. The larger the diameter, the lower the fracture resistance [65]. This is probably due to the additional dentin that must be removed to make room for a thicker post. 

In canals that are ideally shaped, data suggest that the amount of space filled with cement does not affect bond strength; however, this is not the case with root canals that are wide and flared. The high configuration cavity factor (C-factor) within the canal and the shrinkage of polymers in a thick layer of cement can lead to the formation of gaps. Gaps can form along the interface of cement and post or cement and dentin [66]. Moreover, a thick layer of cement increases the likelihood that bubbles or voids will form during application [66].

Solutions have been proposed to overcome the aforementioned issues by reducing the cement gap to a minimum and customizing the post to best fit the root canal shape. These include relining fiber posts with resins or fibers, as well as the use of additional auxiliary posts. It was proposed to use a relining technique to customize the post with RBC to fit the canal’s shape, resulting in minimum cement thickness. This is advantageous for several reasons, including the retention of the post, the improvement of tooth fracture strength, and the reduction of stress transferred to the surface of the cervical root [66].

## 6. Current Advancements and Future Trends

### Bonding Considerations of FRC to Root Canal

The fundamental principles of adhesive bonding involve the use of etchant to create micromechanical adhesion between composite restorations and enamel, as well as the development of an interdiffusion interface between bonding resin and dentine. These techniques are commonly used on teeth with vital pulps and root-filled teeth. The use of a three-step dentine bonding system is the current preferred method for achieving long-lasting dentin bonding [67]. It involves applying a hydrophilic primer to etched dentin to permeate the dentinal tubules and collagen fibrils, followed by using a hydrophobic adhesive comprising Bis-GMA and triethylene glycol dimethacrylate TEGDMA [68]. However, these techniques require careful application, as over-drying the dentin can cause the collagen fibrils to collapse, meaning that the resin cannot penetrate the hybrid layer [69].

If resin infiltration within the hybrid layer is insufficient, it can result in marginal discoloration, hybrid layer degradation, microleakage, and secondary caries [70]. This lack of infiltration may also initiate the re-infection of root-filled teeth. The use of so-called fifth-generation adhesive systems, which combine the primer and adhesive in a single solution to simplify the bonding process by reducing it to two rather than three steps, is a common practice today. However, this technique seems to result in early degradation of the hybrid layer [70,71].

The most recent types of adhesives are referred to as ‘universal’ or ‘all-in-one’ adhesives. These adhesives can perform the functions of etchant, primer, and adhesive within the same solution, and they are compatible with phosphoric acid etching pre-treatment and are suitable for use as self-etching adhesives [72,73]. In conjunction with self-etching primers, these adhesives are commonly used to bond composite restorations and cores in teeth with root canal fillings as well as fiber and metal posts.

To our knowledge, no alterations have been made to the bonding strategy in recent years, even though changes to dentin and enamel substrates have been reported to result in loss of vitality or the need for root canal treatment, such as loss of free water content [74] and collagen alteration [75,76]. These changes are believed to heighten the risk of fractures in root-filled teeth.

Air-abrasion systems with materials such as bioactive glasses or aluminum oxide can effectively prim dentine for restoration after endodontic treatment, as it removes residues and ensures a smooth dentine surface. The lower C-factor decreases the amount of stress along the bonding interface [77,78], thereby decreasing the probability of fatigue failure and crack propagation [79]. Additionally, air abrasion with bioactive glass can create a “bio-reactive” smear layer on the surface of the dentine; this layer can then become an integrated part of the bonding interface created using resin-modified glass ionomer cement or SE adhesives.

The biologically active nature of bioglasses stems from the formation of hydrated silica Si(OH)_4_ when they come into contact with saliva or water. This process can potentially halt degradation at the bonding interface [80,81]. The reactive layer combines with the demineralized dentine collagen [82], solidifying dentine proteases such as metalloproteinases (MMPs) and providing a framework for Ca2+ and PO43− precipitation. This may facilitate remineralization and safeguard the hybrid layer. Additionally, the bioactive glasses’ ability to create an alkaline pH may have antibacterial properties and decrease the likelihood of secondary caries [83].

Alternative techniques proposed for maintaining the longevity of dentin-bound interfaces involve applying chlorhexidine (2% CHX) to acid-etched dentin for one minute prior to bonding. Research conducted both in laboratory settings [84] and on living organisms [85] demonstrates that CHX can inhibit the activity of various forms of MMPs as well as dentinal cysteine cathepsins, resulting in the degradation of the hybrid layer [86].

Although the protocols used for bonding to root canal dentin are similar to those used for coronal restorations, the bond achieved is typically weaker than the bond achieved with coronal dentin [87,88]. The procedure can be challenging due to several factors, such as limited control over moisture levels [88], limited visibility and access, the irregular structural features of secondary dentin, and the small number of dentinal tubules in the apical third of the root [89]. In addition, the level of challenge can be increased by previous preparation procedures or endodontic treatments, which can leave remnants of plasticized gutta-percha and sealer [89], and the fact that some dentin adhesives and resin cement are incompatible [90]. While it may be possible to mitigate some of these challenges, nothing can be done about the very high configuration factor of the endodontic cavity [91]. Consequently, the stresses experienced during the polymerization process may be sufficient to break or weaken the bond between the root canal dentin and the luting material, increasing the likelihood of microleakage and reducing retention [92].

One common way to assess dentin bonding is by measuring bond strength in vitro [93]. Measurement methods include push-out and pull-out tests or measuring micro-tensile strength. However, bond strength is not a material property but rather depends on factors such as the stress rate, the size geometry of the specimen, the type of composite used, and the test method. It is better to perform the push-out test to assess the bond strength of adhesively luted fiber posts as it is closest to clinical conditions [94].

It is essential to evaluate the bond strength and failure mode (location of failure) for each specimen. There are three main types of failure modes cohesive within the test material or tooth substrate, adhesive within the adhesive interface, and mixed. On the same surface, there has been both a cohesive and an adhesive failure [95]. The most common method for determining failure is the use of stereomicroscopes at high magnification, but scanning electron microscopy (SEM) or tandem scanning microscopy (TSM) can provide more accurate information about adhesive and mixed failure modes [95]. A strong correlation exists between the bond strength and the failure pattern–cohesive failure is more prevalent with a high bond strength value [96]. Some studies have used the results of confocal laser scanning microscopy (CLSM) and SEM to predict the durability of the dentin/adhesive interface and how these changes with differing adhesive strategies inside the root canal [97,98,99]. Other microleakage and nanoleakage test methods employing organic dyes as tracers have also been used to evaluate the ability of the dental adhesive to create a seal [100]. However, the microleakage evaluation methods have limited accuracy and sensitivity due to their qualitative nature, which means there may only be insignificant differences in the results of different study groups, making it difficult to interpret the results [101].

Several studies have highlighted the conflicting in vitro studies results obtained when assessing the bonding to root canal dentin [102,103,104,105]. The “self-etch” technique has been reported to be less sensitive than others for root canal adhesion procedures [106]. Another factor to consider when assessing the durability of a bond to the root canal is the degree of how intrinsically the components in the hybrid layer are susceptible to degradation [107]. However, further research is still needed into the exact mechanism of degradation. Reis et al. [108] hypothesized that when highly hydrophilic dental bonding is used (as with self-etching), the water sorption and polymer plasticization will increase, forming a permeable hybrid layer interface. This type of interface has weak mechanical properties [109], and therefore, the bond strength will decrease with time [110]. It is believed that matrix metalloproteinases (MMPs) derived from dentin accelerate the degradation of collagen matrices in aged resin-dentin bonds [111]. Furthermore, Arrais et al. [112] reported a chemical incompatibility in dual-cured resin cement utilized in post-cementation between the acidic monomers of self-adhesive systems and the self-curing components. This incompatibility may account for the variation in results.

## 7. Resin-Based Luting Cements

Resin-based luting cement can be generally defined as low-viscosity composite materials that comprise a dimethacrylate and oligomer monomer matrix. The filler contents ensure low film thickness. Several monomers with different molecular weights, such as UDMA, Bis-GMA, Bis-EMA, and TEGDMA, are combined to produce a material with a high degree of polymerization and, thus, low shrinkage. Silica fillers and silanated radiopaque glass (zirconia, strontium, or barium) are typically used in concentrations ranging from 30 to 60 percent by volume, with particle sizes ranging from 0.5 to 8.0 μm [113].

It is not recommended to use light-cured materials for luting posts, as light has only limited access to the full post space, even if a translucent post is employed. It can, therefore, not be guaranteed that enough light will reach all of the luting material, resulting in insufficient polymerization in some areas [114]. Self-cured cement is preferred as it is set slower than light-cured cement, reducing polymerization stress [115], but it has a shorter working time, making handling more challenging. Dual-cure materials are popular because they combine the strong points of both of the above materials. They are easier to handle as they have a longer working time and continue to polymerize in the deepest parts of the canal. The mechanical properties of dual-curing resins are also higher, as is their polymerization efficacy [115,116,117]. Nonetheless, there is still a lack of consensus over how effective the photo-polymerization of dual-cured cement is. Hashimoto et al. [118] maintain that maximum hardening cannot be achieved with the self-curing mechanism because the chemical activator can have a limited effect on the polymerization of certain dual-cured cement [119,120].

Systems for resin luting can be either conventional or self-adhesive. In the former system, the cement is employed after the application of an adhesive (self-etching or etch-and-rinse primers), whereas self-adhesive resin cement, as the name suggests, does not need the application of an adhesive in order to adhere to the tooth substrate. In terms of their composition, the principal difference is that self-adhesive cement contains acid-functionalized methacrylate monomers, principally phosphoric acid or carboxylic groups. Therefore, they can complete the processes of demineralization and bonding to the tooth substrate in a single step that does not involve a separate adhesive [121].

As previously stated, self-adhesive resin cement is widely used in clinical practice for bonding fiber posts because it reduces nano-leakage and provides a strong bond [122]. It has been suggested that their bond strength could be improved even further with the use of phosphoric acid to pre-condition the surface of the root canal dentin. This would dissolve the thick smear layer and enhance the strength of the bond [123,124]. Conversely, self-adhesive cement is not recommended for building up the core beneath glass ceramic crowns because hygroscopic expansion can damage the crown [125].

One crucial factor to consider is the use of materials based on eugenol for root canal sealers or temporary substances. Eugenol has been shown to have a negative impact on the adhesion between dentine and resin compounds [126,127]. It can also hinder the polymerization process of resin composites by inhibiting the addition of free radicals and penetrating the dentine of root canals [128]. In addition to ultrasonics, air abrasion can be used to prepare the dentine substrate for bonding, as mentioned previously. If materials based on eugenol have been utilized, the dentine can be rinsed with isopropyl alcohol to help reduce the inhibition of composite resin polymerization by sequestering any free eugenol [129].

Due to the restricted access for both the transmission of light and the placing of the restoration, it can be difficult to apply composite resin materials in the root canal and pulp chamber. This can result in the formation of gaps and/or incomplete hardening of the composite resin, which becomes even more challenging if a minimally invasive technique is desired. Research has highlighted that teeth with small access cavities are most prone to voids in composite restorations [130,131]. These studies have also shown that using bulk-fill composites instead of traditional ones decreases the formation of gaps. Bulk-fill materials have been developing rapidly in recent years, and they can considerably shorten the time required to complete a procedure, as their producers claim that they can be applied in layers of up to 10 mm.

Numerous techniques have been implemented by manufacturers of bulk-fill materials to facilitate the placement of more significant quantities of material. These techniques include decreasing the amount of filler and increasing particle size [132], as well as adding photoinitiators to enhance the cure depth. Additionally, they have been made less prone to shrinking through the addition of shrinkage stress modulators [133]. An example of this is SDR (Dentsply Sirona), which uses a modulator that interacts with the camphorquinone photoinitiator during polymerization to slow down the development of the elasticity modulus. Although bulk-fill materials achieve a good cure depth, it should not be forgotten that the intensity of the curing light decreases as distance increases. There can be a number of millimeters between the light source and the bottom of an endodontic cavity; therefore, smaller initial increments are recommended to compensate for this larger distance [134,135]. Hence, except for dual-cure compositions, increments no larger than 4–5 mm should be employed with bulk-fill composite materials.

## 8. One-Stage Procedure: Post-and-Core System

Currently, a popular technique for restoring structurally compromised ETT is to utilize dual-cured resin composite materials with a high filler content [136]. Due to their superior mechanical properties compared to resin cement, these materials are excellent candidates for luting fiber posts and core buildup. The “post-and-core system” allows for the simultaneous completion of luting and buildup [137]. This reduces the time required to complete the procedure, minimizes technique sensitivity, and eliminates the possibility that the core material and cement are incompatible [102].

If the root canal is flared, wide, or non-circular in shape, it is impossible to achieve a snug fit with a prefabricated post. Thus, a thick layer of cement is required, but with the resin cement currently in use, its low modulus of elasticity may result in areas subject to high stress loads, thereby becoming weak points [137]. A better technique is to utilize core materials in the cementation of fiber posts, known as a secondary endodontic monoblock. Restorations carried out with this technique are mechanically homogeneous, and the stress is, therefore, evenly distributed throughout the tooth without further drilling, improving its structure preservation [138]. However, Ferrari et al. [139] reported that the high filler content involved when core buildup materials are used for the cementation of fiber posts could negatively affect the strength of the bond, polymerization stress, and microleakage. As previously mentioned, bonding ability can be reduced by the high C-factor of the endodontic cavity [140] and unfavorable application conditions.

### Future Trends

Alternately, we may see a decline in demand for posts in the future, particularly in molars. There is already a trend towards the use of endocrowns, i.e., crowns and large onlays that do not use a post and core system but rather box-preparation and extrusion of the crown or onlay.

The longevity of root-filled teeth is improved by restorations that enhance their structural integrity while preserving the maximum level of tooth structure. Preparing a “ferrule design” for post-endodontic crown restorations is crucial to prevent tooth or root fractures in teeth that have been substantially compromised [41,141,142]. Preferably, a 2 mm ferrule preparation with a biological width of 2–3 mm requires at least 4–5 mm of supra-crestal tooth tissue, but this is not always feasible in clinical practice. Consequently, root-filled teeth that have lost a substantial amount of their structure may need the surgical crown to be lengthened or orthodontic extrusion. However, the former can significantly change the crown-to-root ratio, increasing stress and strain concentrations in the root dentine and potentially compromising the fracture load behavior and long-term reliability of the post-endodontic restoration [143,144,145]. Therefore, endocrowns offer a conservative alternative for restoring root-filled teeth as they do not require post-space or preparation of a ferrule design.

Recently published meta-analyses and systematic reviews revealed that endocrowns provide high rates of success in both molars (72–99%) and premolars (68–100%) over follow-up periods varying between 3 to 19 years [146]. Endocrowns and traditional post-retained crown restorations have exhibited comparable rates in terms of success and longevity [147], suggesting that endocrowns are a trustworthy alternative treatment for root-filled molars and premolars being compromised. However, a specific preparation design and a meticulous adhesion protocol must be followed prior to extrapolating these results. 

It is recommended that a central retention cavity with a depth of 3 mm and a divergence angle of 6–12° be prepared to ensure a homogeneous diffusion of stress [148,149]. The width of the cervical margin width should be a minimum of 2 mm; it can be either flat or slightly beveled [150].

## 9. Conclusions

Root canal anchoring plays a crucial role in tooth restoration and prosthetic treatment and will continue to be a critical issue in the future. Understanding the principles and mechanics behind creating strong interfaces between root canal posts and dentin is essential to ensure long-lasting outcomes for ETT restored with a crown. With a better understanding of these principles, treatment methods using FRC posts have already undergone significant advancements. Further research in this area will contribute to improving and optimizing the use of FRC posts in dental restorations. Table 2 depicts the most important findings from this review.

## Figures and Tables

**Table 1:** Summary of reviewed studies showing failure rate associated with fiber posts in clinical practice.

Study	Year	Sample Size	Brand Name	Post Material	Final Restoration	Failure Rate	Period (Months)
Fredriksson et al. [7]	1998	236	Composipost	Carbon	All ceramic/metal-ceramic crown	2%	27–41 m
Ferrari et al. [8]	2000	100	Composipost	Carbon	All ceramic/metal-ceramic crown	2%	48 m
Mannocci et al. [9]	2002	110	Carbon posts	Carbon	Direct Composite	3.8%	60 m
King et al. [10]	2003	10	Carbon posts	Carbon	All ceramic/metal-ceramic crown	40%	87 m
Hedlund et al. [11]	2003	65	Composipost Endopost	Carbon	All ceramic/metal-ceramic crown	3%	28 m
Naumann et al. [12]	2005	149	FiberKor, ER, Brasseler	Glass	All ceramic/metal-ceramic crown	19.7%	39 m
Schmitter et al. [13]	2007	50	ER, Brasseler	Glass	All ceramic/metal-ceramic crown	6.5%	14 m
Ferrari et al. [14]	2007	985	Composipost EsthetiPost EsthetiPost Plus	Carbon-Quartz	All ceramic/metal-ceramic crown	7–11%	84–132 m
Bitter et al. [15]	2009	32	DT Light Post	Quartz	Direct composite/full crown	7%	36 m
Schmitter et al. [16]	2011	50	ER, Brasseler	Glass	All ceramic/metal-ceramic crown	28.2%	61 m
Naumann et al. [17]	2012	157	Luscent Anchors (Dentatus) DentinPost (Komet)	Glass	All ceramic/metal-ceramic crown	4.6%	120 m
Sterzenbach et al. [18]	2012	46	White Post DC	Glass	All ceramic/metal-ceramic crown	9.8%	84 m
Sarkis-Onofre et al. [19]	2014	72	White Post DC	Glass	All ceramic/metal-ceramic crown	2.9%	36 m
Parisi et al. [20]	2015	114	Light-Post;	Glass	All ceramic/metal-ceramic crown	14.1%	70 m
Cloet et al. [21]	2017	203	Parapost FibreLux	Glass	All ceramic crown	9.2%	60 m
Bergoli et al. [22]	2018	70	White Post DC (FGM)	Glass	All ceramic crown	7.3	37 m

**Table 2:** Summary of the most important findings in this review.

Remaining Dental Tissue	A 2 mm Margin of Healthy Tissue is Considered Adequate to Provide the Ferrule Effect that Protects the Root.
Post Length	The length of the post must be equal to or greater than that of the crown, or two-thirds the length of the root, with a minimum of 4 mm apical seal.
Post width	The larger the diameter, the lower is the fracture resistance of the tooth.
Resin cement	Self or dual-cure resin cements are recommended; such cements are not fully set just after cementation, even if light-polymerization is used. Therefore, a waiting period of 24 h before final tooth preparation is recommended to ensure maximum polymerization and post-retention.

## Data Availability

Not applicable.
